# Publisher Correction: Increasing the reproducibility of fluid biomarker studies in neurodegenerative studies

**DOI:** 10.1038/s41467-020-20693-0

**Published:** 2021-01-04

**Authors:** Niklas Mattsson-Carlgren, Sebastian Palmqvist, Kaj Blennow, Oskar Hansson

**Affiliations:** 1 Clinical Memory Research Unit, Faculty of Medicine, Lund University, Lund, Sweden; 2 Department of Neurology, Skåne University Hospital, Lund University, Lund, Sweden; 3 Wallenberg Center for Molecular Medicine, Lund University, Lund, Sweden; 4 Memory Clinic, Skåne University Hospital, Malmö, Sweden; 5 Clinical Neurochemistry Laboratory, Sahlgrenska University Hospital, Mölndal, Sweden; 6 Institute of Neuroscience and Physiology, University of Gothenburg, Gothenburg, Sweden

**Keywords:** Biomarkers, Dementia, Neurodegenerative diseases

Correction to: *Nature Communications* 10.1038/s41467-020-19957-6, published online 7 December 2020.

The original version of this Article contained errors in Fig. 2. The words “comparisons” and “independent” were spelled incorrectly. This has been corrected in both the PDF and HTML versions of the Article.

